# Hydrophobic Eutectic Solvent-Based Dispersive Liquid-Liquid Microextraction Applied to the Analysis of Pesticides in Wine

**DOI:** 10.3390/molecules27030908

**Published:** 2022-01-28

**Authors:** Chiara Dal Bosco, Francesca Mariani, Alessandra Gentili

**Affiliations:** 1 Department of Chemistry, Sapienza University of Rome, 00185 Rome, Italy; mariani.1702132@studenti.uniroma1.it (F.M.); alessandra.gentili@uniroma1.it (A.G.); 2 Hydro-Eco, Research Centre of Sapienza University, 00161 Rome, Italy

**Keywords:** pesticides, wine, eutectic solvent, LC-MS, dispersive liquid–liquid microextraction

## Abstract

A green solvent-based DLLME/HPLC-MS method for the determination of 19 pesticides in wine samples has been developed. The extractant solvent is a hydrophobic eutectic mixture composed of L-menthol and butylated hydroxytoluene in a molar ratio of 3:1. The endogenous ethanol of wine has been used as dispersive solvent, in order to avoid the solidification of the extracts under 19 °C. The mobile phase composition, the elution gradient and the sample injection volume were optimized in order to make this hydrophobic mixture compatible with conventional reversed phase chromatography and electrospray ionization. The method was validated in matrix, using a wine free from the target compounds. Average recovery as high as 80%, precision between 3 and 14%, and limits of detection and quantification much lower than the maximum residue levels (MRLs) for grapes and wines fixed by the EU regulation, make this multiresidue method fitted for the purpose, with the further advantages of being quick, cheap and in compliance with the green analytical chemistry. From the analysis of 11 commercial wines it was found that just in a bio sample the target compounds were not detectable or lower than quantification limit; as for the other samples, the most widespread and abundant pesticides were methoxyfenozide and boscalid, but their levels were much lower than the relative MRLs.

## 1. Introduction

Insecticides, fungicides, and herbicides, which are generically referred to as pesticides, are essential for preventing many types of pests, diseases and weed species, which can attack grape vines during the growing season and until grapes ripen [1,2,3,4]. Some pesticides applied during the last stages of ripening are stable during the wine-making process and can be found at the same concentration in grapes and wine, especially those that do not have a preferential partition between liquid and solid phase (such as azoxystrobin, dimethoate and pyrimethanil) [1]. Good agricultural practices and the targeted use of agrochemicals can reduce the pesticide residues in grape and wine. In any case, monitoring the agrochemical levels in commercial wines is very important to certify organic agriculture productions and to assess the dietary exposure, which is the basis for establishing or updating the allowed maximum residue levels (MRLs). Since such limits have not been specifically established for pesticide residues in wine, the MRLs set in European Regulation 2005/396/CE [5] for the raw commodity (wine grapes) are generally applied.

Chromatography, both in gas (GC) and in liquid (LC) phases, coupled to mass spectrometry (MS), is the most used technique for monitoring the pesticide residues in foodstuff [6]. A preliminary sample preparation step is usually necessary to reduce interfering compounds, such as organic acids, sugars and phenolic compounds, and to concentrate the final extract. Even in the absence of a specific regulation on the matter, the availability of highly sensitive methods seems particularly interesting for the certification of organic agriculture products. A widely employed method for the extraction and clean-up of food and beverages before chromatographic analysis is represented by QuEChERS (Quick, Easy, Cheap, Effective, Rugged, Safe). Introduced in 2003 by Anastassiades et al. [7], its success is due to the microscale extraction, which reduces the organic solvent consumption, and to the simple and fast procedure. However, the major disadvantage of this technique is the poor enrichment factor (ER), which can lead to higher detection limits, when compared with other techniques [8]. On the other hand, the dispersive liquid–liquid microextraction (DLLME), developed by Rezaee et al., in 2006 [9], is recognized for its simplicity, low cost, and high ER. Thus far, DLLME has been applied for the extraction of a wide range of compounds from wine, such as phenols [10], phthalic acid esters [11], mycotoxins [12] and pesticides [13,14,15,16,17,18,19,20,21]. As far as this last category is concerned, to the best of our knowledge, only four methods applied the DLLME by using a green extraction solvent, such as 1-undecanol [14] and 1-octanol [15], or 1-dodecanol [16] and a hydrophobic eutectic solvent based on thymol and octanoic acid [17]. However, these last two methods have been developed for the extraction of less than five compounds. Moreover, there is a further DLLME which is not properly green because of the use of dichloromethane as an extraction solvent [18].

Usually, a conventional DLLME procedure employs a dispersive solvent to promote a fine dispersion of the extractant into the aqueous sample. The resulting increase of the contact area between the extractant and the sample solution speeds up the mass transfer of the analytes into the organic phase. Although many DLLME methods are still based on the use of toxic organic solvents, one of the current trends in analytical chemistry is their replacement with safer alternatives [22]. In particular, eutectic solvents (ESs), including both the deep (DESs) and the ideal (IESs) ones, as well as low transition temperature mixtures (LTTMs) and ionic liquids (ILs), are widely used to make DLLME an even greener procedure [23,24,25,26,27]. When dealing with highly hydrophobic ESs, the use of the dispersant solvent is not always necessary, but it can conveniently lower the melting point of some ideal mixtures, such as the one composed of L-menthol and butylated hydroxytoluene at a 3 to 1 molar ratio (MEN:BHT (3:1)). L-menthol has been chosen for its natural derivation, absence of toxicity, and tendency to form hydrophobic eutectic mixtures with selected compounds, such as thymol; BHT, which is a hindered phenolic compound used as antioxidant by food, cosmetic, and pharmaceutical industry, has been preferred to thymol for its lower cost and greener character (penalty points calculated by the analytical Eco-Scale are 1 for BHT, and 4 for thymol), as well as for its additional antioxidant value. This IES with marked antioxidant properties has successfully been employed as an extraction solvent to perform a green DLLME of fat-soluble vitamins and carotenoids from fruit juices [27]. The use of ethanol as a dispersive solvent prevented the solidification of the extracts at temperatures lower than 19 °C.

Here, in order to take advantage of the full potential of this IES, which shows affinity for compounds characterized by logP values ≥ 2, we propose its application to the DLLME of pesticides from wine samples. Owing to its endogenous alcoholic content, wine is a particularly convenient matrix which allows one to reduce the consumption of the ethanol used as the dispersive solvent. To this end, the extraction efficiency of MEN:BHT (3:1) has been studied towards 19 pesticides belonging to different chemical classes. The method has been validated on a white wine, free from the target analytes, and its applicability has been demonstrated through the analysis of 11 real samples.

## 2. Results and Discussion

### 2.1. Fine-Tuning of the Extraction Procedure

**Figure 1:**
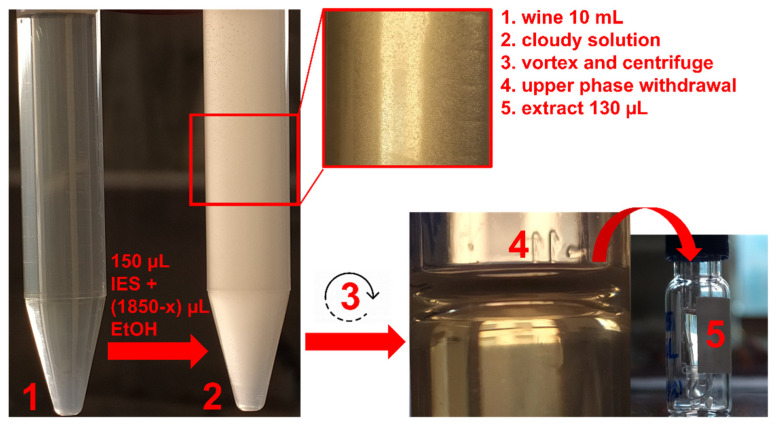
Scheme of the DLLME procedure on wine sample with a certain alcoholic content (x μL of ethanol).

DLLME experiments were performed on the basis of our previous experience with this eutectic solvent, applied to the extraction of fat-soluble micronutrients from fruit juices [27]. In that case, it was found that the best volumes for extracting and dispersing solvents were 150 μL and 1850 μL, respectively. Since ethanol was the dispersing solvent, its endogenous content in wine was exploited for the method optimization described here, in order to reduce solvent consumption. To this end, a sample volume as high as 10 mL was selected. Therefore, the ethanol volume to be added depended on the alcoholic content of the selected sample: for example, for a wine with 13% (*v*/*v*) alcohol, it was 550 μL, i.e., 1850 μL (the total volume)–1300 μL (the endogenous amount). An overall volume of ethanol lower than 1850 μL could not prevent solidification during the extraction and the storage of the extracts at temperatures ≤ 19 °C. This is due to the fact that the liquid state of pure MEN:BHT(3:1) is thermodynamically stable at room temperature and above (≥25 °C) [27], but the partition of ethanol in the eutectic mixture lowers the melting point (up to ≤4 °C). Extractions performed on untreated wine gave an unclear phase separation; therefore, instead of diluting the sample and its useful ethanol content, filtration was preferred as a pretreatment. A scheme of the final DLLME procedure is shown in Figure 1, for details see Section 3.2 and Section 3.3.

### 2.2. Fine-Tuning of the Chromatographic Conditions

In classical DLLME procedures using a high vapor pressure chlorinated solvent, the organic extract is usually evaporated and reconstituted with a solvent system compatible with both the detection system and the used chromatographic conditions, in order to avoid analyte precipitation phenomena and/or peak broadening. On the other hand, when ESs, LTTMs and ILs are used, DLLME extracts are directly injected due to the negligible vapor pressure of such mixtures; therefore, their compatibility with the mobile phase and detectors are crucial requirements. Based on these considerations, we observed that the highly hydrophobic MEN:BHT (3:1) performs very well with non-aqueous reversed phase chromatography (NARP) and atmospheric pressure chemical ionization (APCI) [27]. Nevertheless, we also verified that the studied pesticides were satisfactorily separated by means of conventional reversed phase liquid chromatography (RFLC) and detected with electrospray ionization (ESI) [25,28]. In particular, the RFLC method used a C_18_ column and a mixture of water and acetonitrile as the mobile phase.

**Figure 2:**
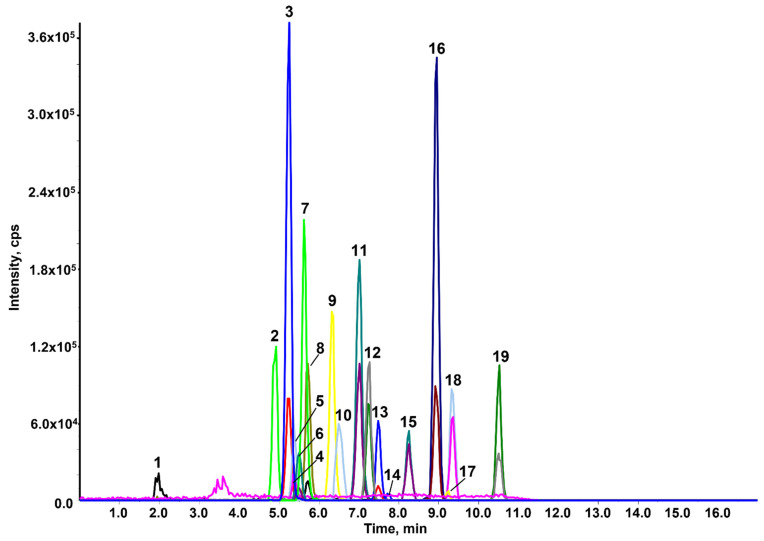
HPLC-MRM of a working standard solution (5 ng injected). Peak legend: 1. DOD, 2. STM, 3. FLD, 4. AZX, 5. MYC, 6. BSC, 7. MXF, 8. TEB, 9. PEN, 10. PRO, 11. BPR, 12. PYR, 13. CLF, 14. CPM, 15. TBF, 16. PPF, 17. CPS, 18. HXT, 19. PRD.

In this study, the RFLC method was carefully modified in order to obtain a good compatibility between the mobile phase composition and the MEN:BHT(3:1) extract, which was directly injected; to this end, the chromatographic conditions and the injection volume were thoughtfully studied. In detail, the solubility of the extract in the mobile phase was evaluated at different percentages of water and acetonitrile: it was found that full solubility was obtained with at least 60% of acetonitrile; therefore, this percentage was set as the initial mobile phase composition. The following gradient elution gave a satisfactory chromatographic separation by increasing acetonitrile to 100% in 9.8 min. Since the extract has an eluotropic strength higher than that of the mobile phase, an injection volume of 2 μL, rather than 5 μL, was the best option for obtaining narrow and symmetric peaks (Figure 2).

A such low injection volume did not exert any negative effect on ESI detection.

### 2.3. Validation Results

Among all the analyzed wines, just one white sample with 13% (*v*/*v*) of alcohol was free from the target analytes (≤LOD); therefore, it was selected as the blank matrix for method validation. It is well known that ESI is a technique prone to a matrix effect that could result in an effect of suppression (very often) or enhancement of the detector signal, providing biased results [29]. Therefore, the matrix effect (ME%) was evaluated for each analyte (Table 1), as described in Section 3.5.

As can be seen, the matrix effect was moderate, for most of the analytes, or negligible, such as for dodine (−2.8%) and penconazole (+1.7%); the highest found value was for clofentezine (−31%). Such results prove that the developed extraction procedure was able to remove major interferences from the final extract; however, due to an average absolute value around 16%, the building of matrix-matched calibration curves is mandatory to make an accurate quantitative analysis. The calibration curves in solvent were also compared with the calibration curves in matrix, obtained from a red wine sample with the minor occurrence of pesticides; the comparison was made only for those analytes for which the wine sample was blank. The ME% resulted comparable with white wine and, for this reason, the matrix-matched calibration curves built from white wine were also used for the quantitative analysis of red wine samples. In order to avoid correcting the concentrations of the unknown positive samples for recoveries, the quantitative analysis was made through the construction of calibration curves in matrix, by spiking the blank aliquots pre-extraction with the analyte standards (see Section 3.5). Table 2 lists the main validation parameters of the DLLME/HPLC-MS method. Section 3.5 describes all the validation procedures in detail.

As can be seen from Table 2, the extraction is characterized by high EFs and recoveries, respectively in the range 43–86 and 56–100%, calculated at a very low spike level of 5 μg L^−1^. The most modest values have been obtained for dodine, as a consequence of its logP lower than 2 (0.96) [30]. The method also stands out for its very good intra- and inter-day precision which is between 3% and 15%. LOD and LOQ values, which vary, respectively, in the ranges 0.00070–1.6 μg L^−1^ and 0.0024–5.0 μg L^−1^, are very low compared with the EU MRLs (see Section 2.5) [5]. The linearity in the studied dynamic range, estimated by means of the least-square method (y = a + bx as regression model), was confirmed by determination coefficients (R^2^) greater than 0.9777 for all the analytes.

### 2.4. Comparison with Other Methods

Table 3 shows a comparison between this work and other three DLLME/GC-MS methods which share some analytes and were developed on alcoholic samples with the use of a traditional chlorinated solvent [13] or a greener one [14,15]. Overall, this work performs equal to or better than either of the others, because high recoveries have been obtained at half spiking level, with very low precision and LOD/LOQ values. The same consideration is even more valid in the comparison with a QuEChERS/GC-MS method [31], also reported in Table 3, in which a ten-fold higher spiking level is used.

### 2.5. Results on Real Samples

**Figure 3:**
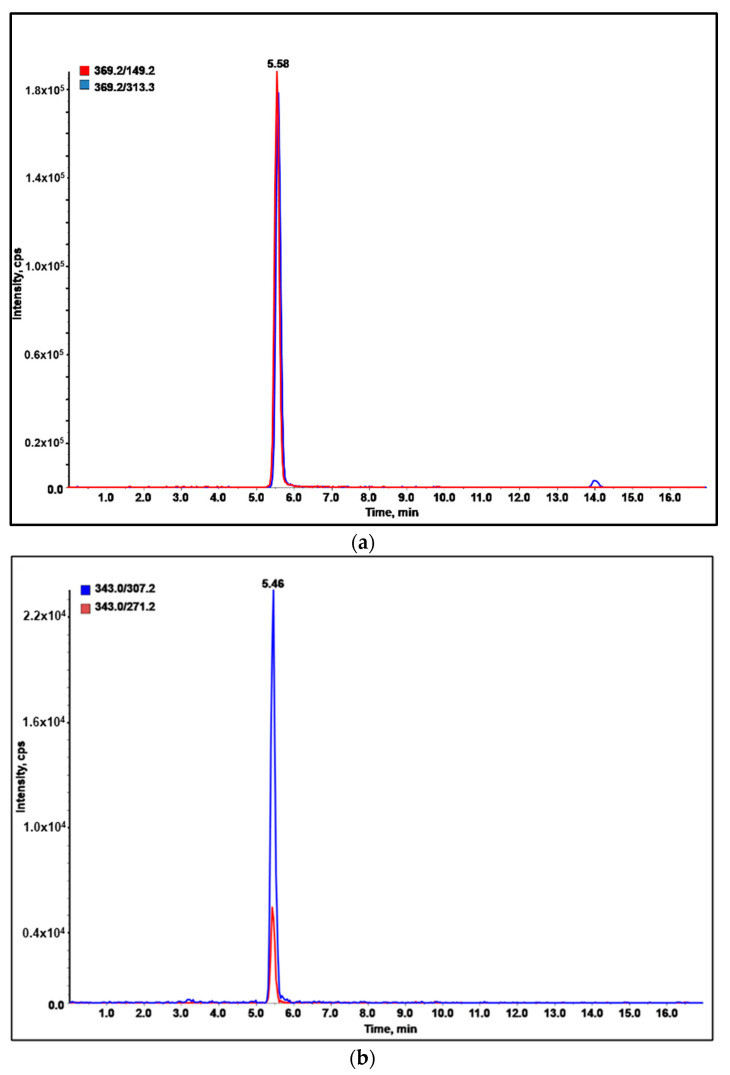
LC-MRM profile of: (**a**) methoxyfenozide in rosé wine; (**b**) boscalid in Prosecco wine.

The quantitative analysis results on eleven commercial wines are reported in Table 4. Only in one biological white wine were the target compounds not detectable or present at concentration lower than the LOD/LOQ of the method. This applied also for most of the compounds in the other samples, in which the more frequently detected and abundant analytes were methoxyfenozide (Figure 3a) and boscalid (Figure 3b). However, all the analyzed samples were in compliance with the European regulation, since the concentrations of the detected pesticides were far below the MRLs.

## 3. Materials and Methods

### 3.1. Chemicals, Materials and Solutions

L-menthol (natural source, food grade, ≥ 99% purity), BHT (food grade, ≥ 99% purity), formic acid, elevated purity grade solvents (acetonitrile, ethanol), as well as analytical standards of azoxystrobin, boscalid, buprofezin, chlorpyrifos, chlorpyrifos-methyl, clofentezine, dodine, fludioxonil, hexythiazox, methoxyfenozide, myclobutanil, penconazole, propiconazole, pyraclostrobin, pyriproxyfen, pyridaben, spirotetramat, tebuconazole and tebufenpyrad were purchased from Sigma Aldrich-Merck S.r.l. (Milan, Italy). A Milli-Q Plus apparatus (Millipore, Bedford, MA, USA.) was used for obtaining ultrapure water.

Weighted amounts of the analytical standards (OhausDV215CD Discovery semi-micro and analytical balance, 81/210 g capacity, 0.01/0.1 mg readability, Ohaus Corporation, Pine Brook, NJ, USA) were dissolved in methanol or toluene (clofentezine and pyraclostrobin) in volumetric flasks, in order to obtain individual stock solutions at a concentration of 1 mg mL^−1^.The last ones were diluted in methanol for preparing the multi-standard working solutions at 0.02, 0.5, 1.5, 2.5 and 4 ng μL^−1^ used for the method validation.

### 3.2. Wine Samples

A total of 11 wines, belonging to different types (6 sparkling or still white wines, 4 red wines, 1 rosé wine) and different geographic areas (with the exception of one sample from South Africa, all other samples were from Italian regions including Friuli Venezia Giulia, Veneto, Umbria, Lazio, Abruzzo and Puglia), were bought in local supermarkets (Rome, Italy). Among both white and red wines, 2 for each group were produced according to the biological agriculture regulation. The alcohol content was in the range 10.5–15% (*v*/*v*). The samples were stored at 4 °C. Before the analysis they were filtered through a 0.45 μm PVDF syringe filter (Sigma-Aldrich, Milan, Italy) and, in the case of sparkling wines, sonicated for 10 min. A white bio sample, in which the studied analytes were not detected or present at their LOD concentrations, was considered as a blank matrix and used for the method validation.

### 3.3. Preparation of the Eutectic Mixture and Extraction Procedure

The extraction solvent composed of MEN:BHT (3:1) was prepared as described in a previous study of ours [24]. DLLME was performed into a polypropylene 15-mL test tube using 10 mL of wine sample. The extraction solvent (150 μL) was mixed with ethanol (1.85 mL), used as the dispersing solvent. For the latter, the volume to add (x mL) was calculated by considering the alcoholic endogenous content of the analyzed sample:

x=V(total)−V(sample)×w

where *x* is the volume of ethanol to add (mL), *V*(*total*) is the total volume of ethanol (1.85 mL), *V*(*sample*) is the volume of wine sample (10 mL), and *w* is the ethanol content in the wine sample.

The mixture was rapidly injected into the sample with a syringe and then the test tube was vortexed for 2 min and centrifugated (6000 rpm, 25 °C) for 5 min. The resulting upper phase was collected with a syringe and directly injected (2 μL) into the HPLC-MS system.

### 3.4. HPLC-MS Analysis

The analysis was performed with a Perkin Elmer series 200 binary pump equipped with an autosampler (Perkin Elmer, Norwalk, CT, USA) and a PE-Sciex API-3000^®^ (Perkin Elmer Sciex Toronto, ON, Canada) triple quadrupole mass spectrometer. The analytes were detected in positive ESI mode by using the following conditions: capillary voltage +4500 V, high purity nitrogen as collision and curtain gas, air as nebulizer and drying gas (350 °C). A polypropylene glycol solution was infused at 10 μL min^−1^ for calibrating at unit resolution each mass-resolving quadrupole, by setting the full width at half maximum (FWHM) at m/z 0.7 ± 0.1. Two multiple-reaction monitoring (MRM) transitions were selected per analyte, the most intense one (quantifier) was used for the quantitative analysis, while the other one (qualifier) was taken as a confirmation criterion in the qualitative analysis.

The analytes were separated on a XTerra C18 column (4.6 × 250 mm, 5 μm), protected by a guard column (Waters, Milford, MA, USA), under gradient elution of water (phase A) and AcCN (phase B), both 10 mM in formic acid. The flow rate of 1 mL min^−1^ was splitted post-column so that just 200 μL min^−1^ were introduced into the ESI source. The gradient was as follows: the column was equilibrated for 5 min at 60% B, which was increased to 100% in 9.8 min and kept the same for 7.2 min. Due to the relatively high viscosity of the extract, a very low-sample speed injection was set for the autosampler needle, which was washed with acetonitrile after each injection. Data were processed by Analyst^®^ 1.5 Software (AB Sciex, Foster City, CA, USA).

### 3.5. Method Validation

The DLLME-LC/MS method was validated in matrix by following the main FDA guidelines for the bioanalytical methods [32]. Recovery, precision, accuracy, sensitivity, linearity, enrichment factor, LODs and LOQs were estimated on a white biological wine whose analyte content was ≤LODs. LODs and LOQs were estimated by injecting extracted samples spiked at decreasing concentrations, until a signal to noise ratio (S/N) of 3 (LOD) or 10 (LOQ) was reached. Accuracy of the proposed methodology was evaluated in terms of recoveries. Mean recoveries and their relative standard deviations on five replicated analyses performed within the same day or one week (intra-/inter- day precisions) were evaluated at a concentration of 5 μg L^−1^, which corresponds to the highest LOQ value, obtained for fludioxonil. Recoveries were calculated as the percentage ratio of the areas obtained from pre-extraction spiked samples compared with those obtained from post-extraction spiked samples. At the same spiking level, the enrichment factor (EF) was evaluated by considering the analyte concentration in the final extract and that in the wine sample, according to the following equation:

$$EF=\frac{C\left(extract\right)}{C\;\left(sample\right)}$$

where *C*(*sample*) is the concentration of the target analyte in the pre-extraction spiked sample, and *C*(*extract*) is the concentration of the same analyte in the final extract.

The quantitative analysis on real samples was performed by means of the pre-extraction external calibration curves built on seven blank aliquots, at the following spike levels: 0.05, 1, 3, 5, 8, 12, and 16 μg L^−1^. At the same levels, a calibration curve in solvent (ethanol) and a post-extraction spiked one in matrix were also built and compared for each analyte, in order to evaluate the matrix effect (ME%) according to the following equation:

$$ME\%=\;\frac{b\left(matrix\right)-b\left(solvent\right)\;\;}{b\left(solvent\right)}\times \;100$$

where *b*(*matrix*) is the slope of the matrix-matched calibration curve, and *b*(*solvent*) is the slope of the analyte curve in solvent.

All calibration curves were built from extractions replicated eight times. Means and standard deviations, linear regression analysis and determination coefficients were calculated with Microsoft Excel 2010 (Microsoft Corporation, Redmond, WA, USA).

## 4. Conclusions

This paper reports the use of the hydrophobic eutectic solvent MEN:BHT (3:1) as extractant for the DLLME of pesticides from wine samples. Within this application, to the best of our knowledge, this is the only method that employs a green extractant solvent combined with a green dispersant solvent, such as ethanol, while at the same time minimizing its consumption by taking advantage of the alcoholic endogenous content of wine. The latter is necessary to maintain the IES in its liquid state at temperatures lower than 19 °C. From this perspective, this method can also be considered an indirect screening test of the alcoholic content in fraud identification, useful to select suspect samples that require further analysis. This multiresidue method, which, to the best of our knowledge, is the only one that combines a green solvent-based DLLME with the LC-MS analysis, has been validated in a matrix for 19 pesticides belonging to different chemical classes: it is characterized by average recovery as high as 80%, precision between 3% and 14%, and LODs and LOQs much lower than the maximum residue levels fixed by the EU regulation for grapes and wines. Therefore, it is suitable for monitoring the pesticide levels in commercial wines from both conventional and biological agriculture. As expected, among the analyzed samples, the biological ones presented the lowest concentrations of the target analytes. Overall, the most widespread and abundant pesticides were methoxyfenozide and boscalid, but their contents were far below the legal limits in all the analyzed samples. In conclusion, the here proposed method is quick, cheap, fitted for the purpose and in compliance with the green analytical chemistry principles.

## Figures and Tables

**Table 1:** Calibration data for the analyte curves in solvent (ethanol), for the analyte curves in matrix by spiking post-extraction, and evaluation of the matrix effect percentage.

Analyte ^a^	Regression Equation (n = 8)		R^2 b^	Matrix Effect %
	b ± S_b_·t_(0.05;6)_	a ± S_a_·t_(0.05;6)_		
AZX				−7.3
Solvent	206.10 ± 10.70	25.70 ± 1.40	0.9894	
Matrix	191.40 ± 12.70	10.20 ± 0.70	0.9992	
BSC				−19
Solvent	36.40 ± 5.30	2.67 ± 0.29	0.9931	
Matrix	29.50 ± 2.90	0.42 ± 0.05	0.9964	
BPR				21
Solvent	9.79 ± 1.23	4.63 ± 0.55	0.9583	
Matrix	11.80 ± 0.89	7.98 ± 0.88	0.8908	
CPS				14
Solvent	2.98 ± 0.45	-	0.9989	
Matrix	3.11 ± 0.47	-	0.9992	
CPM				18
Solvent	2.79 ± 0.39	0.21 ±0.03	0.977	
Matrix	3.29 ± 0.48	−0.52 ± 0.08	0.9673	
CLF				−31
Solvent	33.50 ± 4.20	4.35 ± 0.66	0.9993	
Matrix	23.20 ± 3.40	8.88 ± 1.29	0.9712	
DOD				−2.8
Solvent	21.50 ± 1.10	1.09 ±0.05	0.9982	
Matrix	20.90 ± 0.90	1.89 ± 0.08	0.9972	
FLD				−20
Solvent	0.35 ± 0.04	-	0.9375	
Matrix	0.28 ± 0.03	-	0.9693	
HXT				−22
Solvent	47.80 ± 3.10	−2.94 ± 0.17	0.9994	
Matrix	37.10 ± 2.60	−14.30 ± 0.80	0.9779	
MXF				−15
Solvent	165.50 ± 14.90	36.50 ± 3.30	0.9885	
Matrix	141.00 ± 11.30	4.45 ± 0.41	0.9993	
MYC				−13
Solvent	47.20 ± 2.40	0.14 ± 0.01	0.9924	
Matrix	40.90 ± 2.50	−8.90 ± 0.50	0.9921	
PEN				1.7
Solvent	12.10 ± 1.30	12.30 ± 1.40	0.9058	
Matrix	12.30 ± 1.50	−6.37 ± 0.77	0.8661	
PYR				−12
Solvent	64.10 ± 8.30	0.93 ± 0.12	0.995	
Matrix	56.50 ± 7.30	−13.50 ± 1.70	0.9923	
PRD				−26
Solvent	115.10 ± 16.30	−2.90 ± 0.42	0.9971	
Matrix	84.90 ± 12.40	−21.50 ± 3.10	0.9912	
PPF				18
Solvent	84.40 ± 12.70	−2.88 ± 0.43	0.9869	
Matrix	100.20 ± 15.20	27.40 ± 4.10	0.9961	
PRO				18
Solvent	46.00 ± 5.80	−8.38 ±1.28	0.9938	
Matrix	37.70 ± 4.90	6.72 ± 1.04	0.9803	
STM				−15
Solvent	58.30 ± 5.80	−27.40 ± 3.01	0.957	
Matrix	49.60 ± 4.80	−38.50 ± 4.20	0.9299	
TEB				−15
Solvent	88.20 ± 7.10	0.73 ± 0.06	0.9958	
Matrix	74.80 ± 5.80	−11.00 ± 0.80	0.9978	
TBF				−22
Solvent	23.30 ± 1.50	−4.50 ± 0.27	0.997	
Matrix	18.10 ± 0.90	7.56 ± 0.42	0.9902	

^a^ Abbreviations: AZX = azoxystrobin, BSC = boscalid, BPR = buprofezin, CPS = chlorpyrifos, CPM = chlorpyrifos-methyl, CLF = clofentezine, DOD = dodine, FLD = fludioxonil, HXT = hexythiazox, MXF = methoxyfenozide, MYC = myclobutanil, PEN = penconazole, PRO = propiconazole, PYR = pyraclostrobin, PPF = pyriproxyfen, PRD = pyridaben, STM = spirotetramat, TEB = tebuconazole, TBF = tebufenpyrad. ^b^ Concentration levels ranging from 0.05 to 16 μg L^−1^.

**Table 2:** Main figures of merit of the validated DLLME/HPLC-MS method.

Analyte ^a^	Enrichment Factor ^b^	Recovery ^c^ (%)	Intra-Day Precision ^d^ (RSD%)	Inter-Day Precision ^d^ (RSD%)	Determination Coefficient ^e^ (R^2^)	LOD ^b^ (µg L^−1^)	LOQ ^b^ (µg L^−1^)
AZX	60	78	8	11	0.9968	0.00070	0.0024
BSC	60	78	6	6	0.9977	0.0050	0.036
BPR	65	86	5	9	0.9955	0.0097	0.032
CPS	59	77	8	15	0.9992	0.16	0.54
CPM	82	100	8	11	0.9865	1.0	1.5
CLF	63	82	6	10	0.9979	0.014	0.050
DOD	43	56	11	12	0.9805	1.0	3.0
FLD	86	100	14	13	0.9777	1.6	5.0
HXT	62	81	5	9	0.9979	0.0096	0.032
MXF	68	88	4	5	0.9932	0.030	0.15
MYC	57	74	5	5	0.9987	0.016	0.050
PEN	61	79	3	4	0.9970	0.0083	0.028
PYR	64	83	4	6	0.9873	0.0054	0.018
PRD	51	66	4	7	0.9953	0.018	0.060
PPF	58	75	4	7	0.9961	0.0050	0.020
PRO	62	81	3	3	0.9803	0.10	0.34
STM	58	76	6	6	0.9957	0.0097	0.032
TEB	65	84	3	5	0.9985	0.018	0.030
TBF	68	88	6	8	0.9902	0.030	0.22

^a^ Abbreviations: AZX = azoxystrobin, BSC = boscalid, BPR = buprofezin, CPS = chlorpyrifos, CPM = chlorpyrifos-methyl, CLF = clofentezine, DOD = dodine, FLD = fludioxonil, HXT = hexythiazox, MXF = methoxyfenozide, MYC = myclobutanil, PEN = penconazole, PRO = propiconazole, PYR = pyraclostrobin, PPF = pyriproxyfen, PRD = pyridaben, STM = spirotetramat, TEB = tebuconazole, TBF = tebufenpyrad. ^b^ Average values calculated on five replicates. ^c^ Mean of five independent DLLME/HPLC-MS analysis on white wine spiked at 5 µg L^−1^. ^d^ RSD % of five independent analyses performed within the same day (intra-day precision) or within two weeks(inter-day precision). ^e^ Concentration levels ranging from 0.05 to 16 μg L^−1^.

**Table 3:** Comparison of the main figures of merit of some methods involving the extraction of the same target compounds from wine.

Method	Matrix	Common Analytes	Enrichment Factor	Recovery %	Precision (RSD %)	LOD/LOQ#(µgL^−1^)	Type and Volume of Solvents	Ref.
DLLME- GC/MS	ultrapure water with 40% ethanol (5 mL) (5 mL)	CPS	between 15 and 20 for all the analytes	80	11	0.1/0.34	Extr^a^: tetrachloroethane (400 µL) Disp^b^: endogenous ethanol (2000 µL) diluted with water (7.5 mL)	[13]
		CPM		109	2	0.07/0.22		
		MYC		100	11	0.2/0.80		
		TEB		68	9	1.4/4.7		
				(10 µg L^−1^ spike level)				
DLLME- GC/MS	white wine (5 mL)	FLD	66	108	8.2	n.d./0.8	Extr^a^: 1-undecanol (50 µL) Disp^b^: acetone (500 µL)	[14]
		PEN	72	100	6.8	n.d./0.3		
		PRO	71	107	7.1	n.d./1		
		TEB	68	102	6.8	n.d./0.6		
				(10 µg L^−1^ spike level)				
DLLME- GC/MS	white wine (2.5 mL) water diluted to 5 mL	FLD	1254	82	4.3	0.022/0.074	Extr^a^: 1-octanol (11 µL)	[15]
		TEB	1116	74	1.3	0.010/0.032		
				(5 µg L^−1^ spike level)				
QuEChERS-GC/MS	white wine (10 mL)	BPR	n.d.	92	11	n.d./20	Acetonitrile (5 mL) + NaCl (3 g), MgSO_4_ (4 g), PSA (50 mg)	[31]
		CPS	n.d.	84	12	n.d./30		
		CPM	n.d.	93	10	n.d./20		
		PRD	n.d.	87	9	n.d./7		
		PRO	n.d.	91	5	n.d./40		
		TEB	n.d.	83	6	n.d./40		
				(50 µg L^−1^ spike level)				
DLLME- HPLC/MS	white wine (10 mL)	BPR	65	86	5	0.0097/0.032	Extr^a^: MEN:BHT (3:1)(150 µL) Disp^b^: endogenousethanol + addedethanol(total 1850 µL)	This work
		CPS	59	77	8	0.16/0.54		
		CPM	82	100	8	1.0/1.5		
		MYC	57	74	5	0.016/0.050		
		FLD	86	100	14	1.6/5.0		
		PEN	61	79	3	0.0083/0.028		
		PRD	51	66	4	0.018/0.060		
		PRO	62	81	3	0.10/0.34		
		TEB	65	84	3	0.018/0.030		
				(5µg L^−1^ spike level)				

**Table 4:** Pesticide levels in commercial white (Moscato, Prosecco, Chenin Blanc, Pecorino, Sauvignon), rosé and red (Chieti, Montepulciano, Cabernet, Negroamaro) wines.

Analyte	Concentration (µgL^−1^)											
	Moscato	Prosecco 1	Prosecco 2	Chenin Blanc	Pecorino Bio	Sauvignon Bio	Rosé	Chieti Bio	Montepulciano Bio	Cabernet	Negroamaro	MRL
AZX	0.17	1.1	0.16	0.090	n.d.	LOD	<LOD	LOD	LOD	n.d.	0.15	3000
BSC	1.1	4.0	10	1.2	<LOD	LOD	0.59	LOD	LOD	n.d.	0.54	5000
BPR	n.d.	n.d.	n.d.	n.d.	n.d.	n.d.	n.d.	n.d.	n.d.	n.d.	n.d.	10
CPS	n.d.	n.d.	n.d.	n.d.	n.d.	n.d.	n.d.	n.d.	n.d.	n.d.	n.d.	10
CPM	n.d.	n.d.	n.d.	n.d.	n.d.	n.d.	n.d.	n.d.	n.d.	n.d.	n.d.	10
CLF	n.d.	n.d.	n.d.	n.d.	n.d.	n.d.	n.d.	n.d.	n.d.	n.d.	n.d.	1000
DOD	n.d.	n.d.	n.d.	n.d.	n.d.	n.d.	n.d.	n.d.	n.d.	n.d.	n.d.	10
FLD	n.d.	n.d.	n.d.	n.d.	n.d.	n.d.	n.d.	n.d.	n.d.	n.d.	n.d.	4000
HXT	n.d.	n.d.	n.d.	n.d.	n.d.	n.d.	n.d.	n.d.	n.d.	n.d.	n.d.	1000
MXF	2.4	3.9	1.9	<LOD	0.29	n.d.	14.4	0.30	1.5	<LOD	9.6	1000
MYC	0.39	<LOD	LOD	<LOQ	<LOQ	n.d.	n.d.	n.d.	n.d.	0.15	0.36	1500
PEN	LOD	n.d.	n.d.	<LOQ	n.d.	n.d.	LOQ	n.d.	n.d.	n.d.	LOQ	500
PYR	n.d.	n.d.	n.d.	n.d.	n.d.	<LOD	n.d.	n.d.	n.d.	n.d.	n.d.	2000
PRD	n.d.	n.d.	LOD	LOD	<LOD	n.d.	<LOD	LOD	LOD	<LOQ	LOD	10
PPF	n.d.	n.d.	n.d.	n.d.	n.d.	n.d.	n.d.	n.d.	n.d.	n.d.	n.d.	50
PRO	n.d.	n.d.	n.d.	n.d.	n.d.	n.d.	n.d.	n.d.	n.d.	n.d.	n.d.	300
STM	n.d.	n.d.	n.d.	n.d.	n.d.	n.d.	n.d.	n.d.	n.d.	n.d.	n.d.	2000
TEB	0.71	n.d.	<LOQ	2.1	LOD	n.d.	0.060	<LOD	<LOD	<LOD	0.33	1000
TBF	n.d.	n.d.	n.d.	n.d.	n.d.	n.d.	n.d.	n.d.	n.d.	n.d.	n.d.	600

## Data Availability

Not applicable.

## Sample Availability

Samples of the analyzed wines are not available from the authors.
